# Platelet-Derived Factor V Is an Important Determinant of the Metastatic Potential of Circulating Tumor Cells

**DOI:** 10.3389/fonc.2020.558306

**Published:** 2020-09-23

**Authors:** Xin Deng, Ziqian Feng, Luochen Zhu, Ni Chen, Yifei Deng, Yongjie Li, Rong Li, Liqun Wang, Mao Luo, Jianbo Wu

**Affiliations:** ^1^Key Laboratory of Medical Electrophysiology of Ministry of Education, Collaborative Innovation Center for Prevention and Treatment of Cardiovascular Disease of Sichuan Province, Drug Discovery Research Center, Southwest Medical University, Luzhou, China; ^2^Laboratory for Cardiovascular Pharmacology, Department of Pharmacology, School of Pharmacy, Southwest Medical University, Luzhou, China; ^3^Department of Orthopedics, West China Hospital, Sichuan University, Chengdu, China

**Keywords:** platelets, factor V, tissue factor pathway inhibitor, metastasis, coagulation cascade

## Abstract

Factor V (FV) is a critical component in the blood coagulation cascade. In patients, FV inhibitors have been reported to be associated with malignancy. FV is present in plasma and platelets, which exhibit physical and functional differences. However, the functions of FV in cancer progression remain poorly understood. We evaluated the impact of different levels of FV in plasma and platelets on the haematogenous mouse pulmonary metastasis model to determine whether FV determines the metastatic potential of circulating tumor cells. The role of platelet-derived FV was evaluated using a murine B16F10 pulmonary metastasis model, an assay of tumor cell adhesion to endothelial cells, and western blotting. By combining genetic models and FV inhibitory antibody, the transgenic mice with lower platelet FV expression showed significant increases in metastases compared with mice with higher platelet FV expression. *In vitro*, labeled B16F10 melanoma cells appeared to exhibit increased adhesion to endothelial cells that were treated with lower levels of platelet FV, but not platelet-poor plasma. Furthermore, platelets from mice with lower platelet FV levels expressed TFPIα at lower levels than with mice with higher platelet FV expression. Based on these findings, platelet-derived FV contributes to haematogenous pulmonary metastasis and is associated with the regulation of tumor cell adhesion to the vessel wall.

## Introduction

The association between coagulation activation and tumor progression is well documented (1–3). Modulation of the function of platelets and various constituents of the blood coagulation cascade, including thrombin, tissue factor (TF), factor VIIa (FVIIa), factor Xa (FXa), fibrinogen, and vascular cells, has been clearly documented in both *in vitro* and *in vivo* tumor models. A crucial step in cancer metastasis is the survival of cancer cells in the circulation. This process involves the interactions of tumor cells with platelets, shielding them from immune responses in the circulation (4–6). Eventually, the direct interaction of tumor cells with endothelial cells determines their metastatic spread (7).

Factor V (FV) is a critical regulator of the coagulation cascade that involves both procoagulant and anticoagulant pathways. FV functions as a cofactor in the prothrombinase complex, which plays a vital role in the activation of prothrombin to thrombin. FV is present in distinct plasma and platelet compartments, and ∼80% of the circulating FV is derived from plasma, while ∼20% is stored in platelets (8). Based on accumulating evidence, FV in human platelets and plasma has distinct functions (8, 9). Patients with an undetectable plasma level of FV but detectable platelet FV level show relatively mild clinical bleeding, indicating that platelet FV plays a critical role in the regulation of coagulation (10). Importantly, platelet-derived FV functions as a potential mediator of a more procoagulant phenotype (8, 9, 11). Tissue factor pathway inhibitor (TFPI) is a plasma multivalent Kunitz-type serine protease inhibitor that interacts with FV, and this interaction may alter the formation of venous thrombosis (12) and may be related to cancer progression (13).

According to a previous study, single nucleotide polymorphisms (SNPs) in the F5 gene are associated with the progression of breast cancer (14), and a high level of F5 expression is associated with aggressive tumors and survival in patients with breast cancer (15), suggesting a novel role for FV in tumor progression. As shown in our recent study, platelet-derived FV plays an important role in controlling angiogenesis and is likely associated with thrombin activity (16). However, researchers have not yet determined whether the levels of FV are associated with tumor progression and metastasis. Using transgenic mice that express different levels of the FV gene in either plasma or platelets (17), we recently identified a critical role for platelet-derived FV in the regulation of arterial thrombosis through platelet activation (18). In the current study, we evaluated the role of FV from different sources in metastasis.

## Materials and Methods

### Animals

Tg^–^F5^+/+^, Tg^+^F5^+/–^, and Tg^+^F5^–/–^ mice were previously generated on the C57BL/6 background and characterized (17). The mice exhibited different levels of FV gene expression restricted to either the plasma or platelets (Tg^–^F5^+/+^, 100/100%; Tg^+^F5^+/–^, 65/50%; Tg^+^F5^–/–^, 15/0%; Tg indicates transgenic mice. Tg^+^ indicates that the mice carry the transgene, and Tg^–^ represents mice lacking the transgene) (19). These mice were backcrossed onto the C57BL/6J genetic background for six or more generations (≥N6). Male Tg^–^F5^+/+^, Tg^+^F5^+/–^, and Tg^+^F5^–/–^ littermates aged 6–8 weeks were used in all experiments. The experimental procedures were approved by Institutional Animal Care and Use Committee of Southwest Medical University.

### Cell Lines

Murine B16F10 melanoma cells and immortalized mouse brain endothelial cells, bEnd.3, were obtained from the American Type Culture Collection (Manassas, VA, United States) and cultured in Dulbecco’s Modified Eagle Medium (DMEM) supplemented with 10% fetal bovine serum (FBS; HyClone Laboratories, Logan, UT, United States), a 1% penicillin-streptomycin solution, and 2 mM L-glutamine in a humidified incubator containing 5% CO_2_ at 37°C.

### Experimental Pulmonary Metastasis Model

Murine B16F10 cells were resuspended in PBS, and a volume of 200 μl (3 × 10^5^ cells) was intravenously injected into the tail vein of mice. Animals were sacrificed on day 14 after cancer cell inoculation and the lungs were harvested. After fixation with 4% neutral-buffered formalin, the surface of the lungs was examined macroscopically for the presence of metastases.

In some experiments, the B16F10 cells were incubated with anti-FV antibody (50 μg per mouse) (a gift from Dr. David Ginsburg, University of Michigan) or mouse IgG, and inoculated intravenously into the lateral tail vein of male C57BL/6J mice (*n* = 6 mice per group).

### Assay of Tumor Cell Adhesion to Endothelial Cells

The bEnd.3 cells were grown in DMEM supplemented with 15% FCS, and incubated in 24-well plates at a density of 1 × 10^5^ cells/well until the cellular confluence reached 90%. After washes with PBS, bEnd.3 cells were pretreated with equal amounts of platelets or PPP from different FV transgenic mice for 4 h, and then CellTracker^TM^ Green CMFDA–labeled B16F10 cells were added to each well with serum-free DMEM. After 1 h, wells were washed with PBS and adherent fluorescent cells were lysed with RIPA buffer (Sigma-Aldrich, St. Louis, MO, United States). Equal volumes of the cell lysates were measured using a fluorescence microplate reader (Molecular Devices).

For antibody blockade experiments, isolated platelets derived from C57BL/6J mice were pretreated with an anti-mouse FV antibody (10 μg/mL) for 6 h before a co-incubation with CMFDA-labeled B16F10 and bEnd.3 cells.

### Immunoblotting

Platelets lysates were prepared as previously described (20), and equal amounts of protein were separated on SDS-PAGE gels and transferred to polyvinylidene difluoride membranes using electroblotting. After blocking, the membranes were incubated with antibodies against mouse TFPI and GAPDH. ImageJ software (National Institutes of Health, Bethesda, MD, United States) was used to quantify band densities.

#### Plasma FV Antigen Measurement

Plasma samples were prepared by centrifugation. The level of the FV antigen was measured using a mouse coagulation FV ELISA kit (Cusabio Technology LLC, Houston, TX).

### Statistical Analysis

Data are presented as the means ± standard errors of the means. Experimental groups were compared using two-tailed Student’s *t*-test or one-way analysis of variance (ANOVA). The level of significance was set to *P* < 0.05.

## Results and Discussion

**FIGURE 1:**
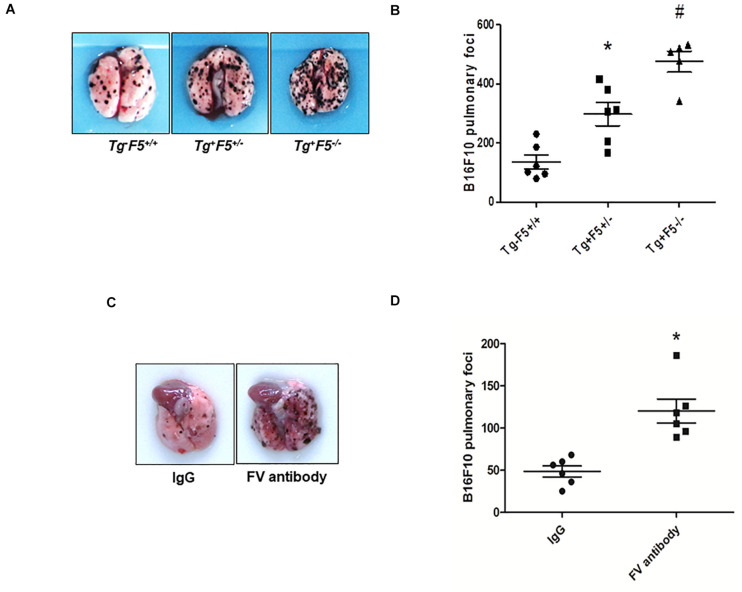
Platelet-derived FV regulates tumor metastasis. **(A)** B16F10 tumor cells were injected in the indicated mice. Numbers of metastatic foci on the lung surface of B16F10-injected Tg^–^F5^+/+^, Tg^+^F5^+/–^, and Tg^+^F5^–/–^ mice were counted. Representative photographs of pulmonary metastatic foci produced 14 days after the intravenous injection of B16F10 cells (*n* = 9, 7, or 5) are shown. **(B)** Results of the quantitative analysis of pulmonary metastatic foci. *P < 0.05 compared with Tg^–^F5^+/+^ mice. ^#^*P* < 0.05 compared with Tg^–^F5^+/+^ and Tg^+^F5^+/–^ mice (*n* = 6–9 mice per group). **(C)** Effect of the FV antibody on an experimental metastasis model with C57BL/6J mice *in vivo*. Representative photographs of pulmonary metastatic foci produced 14 days after the intravenous injection of B16F10 cells treated with an anti-FV antibody (50 μg per mouse) or mouse IgG. **(D)** Results of the quantitative analysis of pulmonary metastatic foci. *P < 0.05 compared with the IgG group (*n* = 6 mice per group).

**FIGURE 2:**
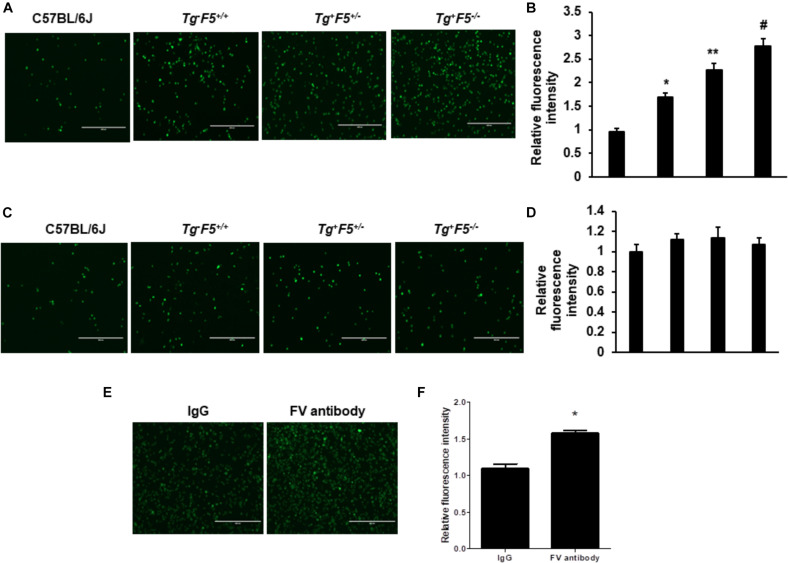
Effect of platelet-derived FV on tumor cell adhesion to endothelial cells *in vitro*. **(A)** Effects of platelets on the adhesion of CMFDA-labeled B16F10 melanoma cells to bEnd.3 cells, as indicated. The efficiency of the adhesion of B16F10 tumor cells was observed under a fluorescence microscope. **(B)** Results of the quantitative analysis of the adhesion of CMFDA-labeled B16F10 melanoma cells to bEnd.3 cells, as indicated. **(C)** Effect of PPP on tumor cell adhesion to endothelial cells *in vitro*. The adhesion of CMFDA-labeled B16F10 melanoma cells to bEnd.3 cells was assessed, as indicated. **(D)** Results of the quantitative analysis of the adhesion of CMFDA-labeled B16F10 melanoma cells to bEnd.3 cells. Data are presented as the means ± SEM of triplicate experiments. Statistical analysis: one-way ANOVA with Bonferroni’s multiple comparisons test. *P < 0.05 compared with C57BL/6J mice, **P < 0.05 compared with Tg^–^F5^+/+^ mice, and ^#^*P* < 0.05 compared with Tg^–^F5^+/+^ and Tg^+^F5^+/–^ mice. Scale bar: 200 μm. **(E)** Effect of the FV antibody on tumor cell adhesion to endothelial cells *in vitro*. Isolated platelets derived from C57BL/6J mice were pretreated with the anti-mouse FV antibody or IgG (10 μg/mL) for 6 h before the co-incubation with CMFDA-labeled B16F10 and bEnd.3 cells. The efficiency of B16F10 tumor cell adhesion was observed under a fluorescence microscope. **(F)** Results of the quantitative analysis of the adhesion of CMFDA-labeled B16F10 melanoma cells to bEnd.3 cells. *P < 0.05 compared with the IgG group. Data are presented as the means ± SEM of triplicate experiments.

We compared the number of lung metastasis between Tg^–^F5^+/+^, Tg^+^F5^+/–^, and Tg^+^F5^–/–^ mice to determine the effect of platelet-derived FV on metastasis. Fourteen days after cancer cell inoculation, B16F10 melanoma cell colonization was macroscopically visible in the lungs of all animals. The number of lung metastases was increased by 1.2- and 2.3-fold in Tg^+^F5^+/–^ and Tg^+^F5^–/–^ mice, respectively, when compared with Tg^–^F5^+/+^ mice (Figures 1A,B). This result provides evidence for the importance of platelet FV in tumor metastasis. We further investigated the effect of an FV antibody *in vivo* using an experimental metastasis model with C57BL/6J mice. As shown in Figures 1C,D, the B16F10 cells that were co-incubated with the anti-FV antibody produced a markedly increased number of surface pulmonary nodules compared to the control group in which cells were treated with IgG. Based on these data, FV is involved in tumor metastasis *in vivo*. We evaluated the molecular mechanisms by which platelet-derived FV inhibits tumor metastasis. As platelets mediate the interaction of tumor cells with endothelial cells, we examined the adherence of platelets or platelet-poor plasma to endothelial cells using CMFD-labeled B16F10 melanoma cells. As shown in Figures 2A,B, the fluorescence intensity, which corresponded to B16F10 melanoma cell adhered to bEnd.3 cells, was significantly increased after a pretreatment by Tg^+^F5^–/–^-derived platelets compared with platelets from Tg^+^F5^+/–^, Tg^–^F5^+/+^, and WT mice. However, the incubation of PPP with endothelial cells did not produce significant differences in adhesion between the groups (Figures 2C,D). We further investigated whether the inhibitory effect of platelet-derived FV was associated with the adhesion of tumor cells to endothelial cells. As shown in Figures 2E,F, compared with control IgG, the adhesion of B16F10 cells to bEnd.3 cells was significantly increased when platelets were pretreated with the anti-FV antibody. Thus, platelet-derived FV is implicated in decreasing the adhesion of tumor cells to endothelial cells.

**FIGURE 3:**
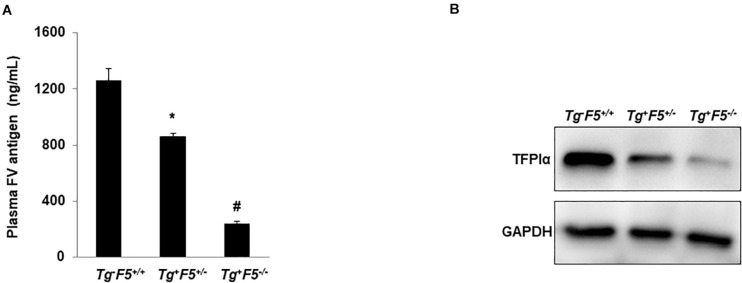
Plasma FV level and TFPIα expression in platelets. **(A)** Plasma levels of the FV antigen in tumor-bearing Tg^–^F5^+/+^, Tg^+^F5^+/–^, and Tg^+^F5^–/–^ mice were measured using an ELISA (*P* < 0.01) (*n* = 6 mice per group); *P < 0.05 compared with Tg^–^F5^+/+^ mice. ^#^*P* < 0.05 compared with Tg^–^F5^+/+^ and Tg^+^F5^+/–^ mice. **(B)** Platelets were isolated from Tg^–^F5^+/+^, Tg^+^F5^+/–^, and Tg^+^F5^–/–^ mice. The lysates of platelets were analyzed using western blotting with a polyclonal anti-TFPIα antibody.

Our previous studies have shown the expression of FV levels in either platelets or PPP from these transgenic mice (Tg^–^F5^+/+^, Tg^+^F5^+/–^, and Tg^+^F5^–/–^ mice). Compared with the Tg^+^F5^+/–^ and Tg^+^F5^–/–^ mice, Tg^–^F5^+/+^ mice showed the highest level of FV expression in both platelets and PPP. The Tg^+^F5^–/–^ mice expresses ∼15% of the wild-type plasma FV level with no detectable platelet FV (16). In the present study, we measured the plasma level of FV in these genetic pulmonary metastasis models. The mice were sacrificed after 14 days and the plasma FV levels were measured using an ELISA. The levels of FV were 1.5- to 3-fold higher in the tumor-bearing Tg^–^F5^+/+^ mice than in tumor-bearing Tg^+^F5^+/–^ and Tg^+^F5^–/–^ mice (*P* < 0.01) (Figure 3A). The difference in FV levels between these genetic pulmonary metastasis models supports the findings of our experiments showing that increased levels of FV reduced haematogenous pulmonary metastasis, and platelet-derived FV inhibited the adhesion of tumor cells to endothelial cells. TFPIα interacts with FV in plasma (21–23). Evidence for the non-haemostatic tumor-suppressive activities of TFPI is accumulating (13). Here, we examined the expression of TFPIα on platelets using western blotting (Figure 3B). Platelet lysates from Tg^+^F5^–/–^ mice expressed TFPIα at lower levels than platelets from Tg^+^F5^+/–^ and Tg^–^F5^+/+^ mice, providing evidence for an additional mechanism by which FV regulates TFPIα expression in platelets. Spiezia et al. (24) reported critical roles for plasma FV in initiating the formation of thrombin and in promoting platelet-dependent thrombin formation that accelerates fibrin formation in patients with a congenital FV deficiency. In addition, the authors found that the addition of normal platelets reduced the time required to initiate clotting, suggesting an important role for platelet FV release in initiating thrombin generation. As shown in our previous study, mice with lower platelet FV levels exhibit slower thrombotic occlusion of the carotid artery after injury (18). However, based on our current findings, mice with lower platelet FV develop more lung metastases due to increased adhesion of tumor cells to endothelial cells. These findings imply that platelet FV might have important non-haemostatic functions. Thus, circulating FV might serve as a valuable biomarker of cancer progression. In the present study, mice lacking FV were more susceptible to cancer cell metastasis after the injection of melanoma cells into the tail vein. This study had some limitations. Platelet-tumor cell interactions induce platelet activation and aggregation. However, the exact role of platelet-derived FV in initiating and modulating the interactions between the platelets, tumor cells, and endothelial cells was not fully defined. Future studies are needed to characterize the contribution of platelet-derived FV to the interactions among platelets, tumor cells, and endothelial cells and the involvement of specific cell surface receptors.

## Data Availability Statement

The raw data supporting the conclusions of this article will be made available by the authors, without undue reservation.

## Ethics Statement

The animal study was reviewed and approved by the animal care committee of Southwest Medical University in accordance with the Institutional Animal Care and use committee guidelines.

## Author Contributions

XD, ZF, LZ, NC, and RL conceived the study, participated in its design, and had full access to all of the data in the study. YD, YL, LW, and ML carried out the animal experiments. XD and JW interpreted the data and wrote the manuscript. All authors were involved in data interpretation.

## Conflict of Interest

The authors declare that the research was conducted in the absence of any commercial or financial relationships that could be construed as a potential conflict of interest.
